# Identification of an Evolutionarily Conserved Cis-Regulatory Element Controlling the Peg3 Imprinted Domain

**DOI:** 10.1371/journal.pone.0075417

**Published:** 2013-09-10

**Authors:** Michelle M. Thiaville, Hana Kim, Wesley D. Frey, Joomyeong Kim

**Affiliations:** Department of Biological Sciences, Louisiana State University, Baton Rouge, Louisiana, United States of America; Harbin Institute of Technology, China

## Abstract

The mammalian Peg3 domain harbors more than 20 evolutionarily conserved regions (ECRs) that are spread over the 250-kb genomic interval. The majority of these ECRs are marked with two histone modifications, H3K4me1 and H3K27ac, suggesting potential roles as distant regulatory elements for the transcription of the nearby imprinted genes. In the current study, the chromatin conformation capture (3C) method was utilized to detect potential interactions of these ECRs with the imprinted genes. According to the results, one region, ECR18, located 200-kb upstream of Peg3 interacts with the two promoter regions of Peg3 and Zim2. The observed interaction is most prominent in brain, but was also detected in testis. Histone modification and DNA methylation on ECR18 show no allele bias, implying that this region is likely functional on both alleles. In vitro assays also reveal ECR18 as a potential enhancer or repressor for the promoter of Peg3. Overall, these results indicate that the promoters of several imprinted genes in the Peg3 domain interact with one evolutionarily conserved region, ECR18, and further suggest that ECR18 may play key roles in the transcription and imprinting control of the Peg3 domain as a distant regulatory element.

## Introduction

In mammals, a small subset of autosomal genes are expressed mainly from one parental allele due to an epigenetic mechanism termed genomic imprinting [1]. These genes are usually clustered in specific regions of chromosomes, wherein the genomic structure of each imprinted domain, e.g. gene order, orientation and distance, is well conserved among mammalian species [1]. The transcription and imprinting of genes in a given domain is controlled through small genomic regions, called Imprinting Control Regions (ICRs) [2]. The mechanisms by which each ICR controls its domain are believed to involve long-range genomic interactions between the ICR and its associated imprinted genes. Consistent with this, disrupting the genomic structure of imprinted domains is known to cause global effects on each imprinted domain, affecting both the allele-specific and spatial and temporal expression patterns of the associated imprinted genes [1–3]. The studies on the H19/Igf2 domain further provide a well-known paradigm that the ICR of this domain functions as an allele-specific insulator that can allow or block long-range interactions between the shared enhancer and the promoters of the two genes, H19 and Igf2 [4,5]. Given the evolutionary constraints often observed in the genomic structures of large imprinted domains, it is likely that similar long-range genomic interactions might have played significant roles in controlling mammalian imprinted domains.

Peg3 (paternally expressed gene 3) was the first imprinted gene identified from the 500-kb genomic interval of proximal mouse chromosome 7 [6]. Subsequently, 6 additional imprinted genes have been identified: three paternally expressed genes (Usp29, Zfp264, APeg3) and three maternally expressed genes (Zim1, Zim2, Zim3) [7–12]. As seen in other imprinted domains, this domain has also been well preserved during mammalian evolution. The evolutionary conservation of this genomic interval is in stark contrast to the lineage-specific expansion and/or shrinking of the two gene families surrounding the Peg3 domain, Olfactory receptor (OLFR) and Vomeronasal organ receptor (VNO) gene families [13,14]. Interestingly, in the Peg3 domain, the majority of the imprinted genes except Peg3 have lost their open reading frames independently in different lineages of mammals [14]. Yet, they have maintained their transcriptional activity throughout mammalian evolution, suggesting unusual functional selections on these imprinted genes [14]. Also, according to recent results, this domain is controlled through one ICR, Peg3-DMR (Differentially Methylated Region), which is the 4-kb region surrounding the first exons of Peg3 and Usp29 [15]. Deletion of this ICR results in changes in the allele-specific expression of two genes, Zim2 and Zfp264, and also causes up-regulation in the expression levels of most of the genes in this domain [15]. The actual mechanism by which this ICR controls the entire domain is currently unknown, but we predict that this unknown mechanism is likely mediated through long-range genomic interactions between the identified ICR and unknown *cis*-regulatory elements similar to other imprinted domains [1–5].

The Peg3 domain is known to be controlled through two *cis*-regulatory elements, Conserved Sequence Element (CSE) 1 and 2, which are located within the Peg3-DMR [16]. CSE1 is a strong repressor for the transcription of Peg3 and Usp29, however the factors binding to CSE1 are currently unknown [16,17]. In contrast, CSE2 is a DNA-binding site for YY1, which is thought to be involved in epigenetic setting and/or maintenance of the Peg3-DMR [18–21]. Besides these two *cis*-regulatory elements, the Peg3 domain also contains a large number of small genomic regions that are evolutionarily conserved according to previous comparative genomic analyses [7,14]. Interestingly, recent genome-wide ChIP surveys indicate that many of these evolutionarily conserved regions (ECRs) are marked with two histone modifications, H3K4me1 (monomethylation at lysine 4 of histone 3) and H3K27ac (acetylation at lysine 27 of histone 3), suggesting that these regions are likely distant regulatory elements for the Peg3 domain [22]. In the current study, we performed a series of Chromatin Conformation Capture (3C) and Chromatin Immunoprecipitation (ChIP) analyses to further confirm this prediction. Overall, the current study identifies one region, ECR18, as a key regulatory region for the transcription and imprinting of the Peg3 domain.

## Results

### Identification of 18 ECRs

**Figure 1:**
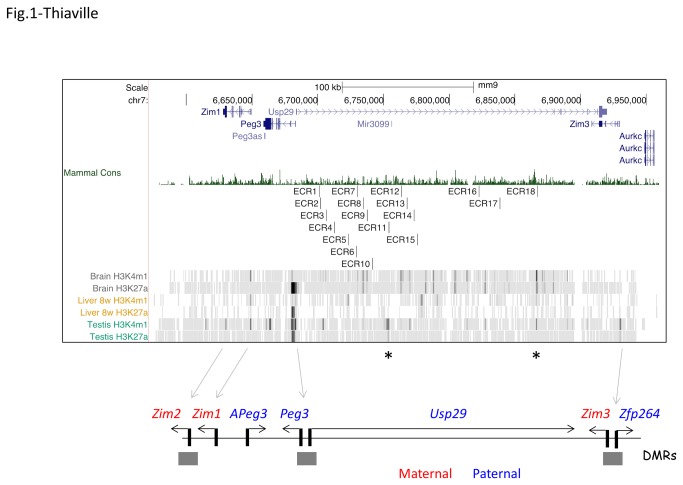
Evolutionarily Conserved Regions (ECRs) in the Peg3 domain. The upper panel represents a snapshot from UCSC Genome Browser showing the chromosomal position of the Peg3 domain, mammalian conservation levels (Mammal Cons), the positions of 18 ECRs, and histone modification profiles of H3K4me1 and H3K27ac observed in brain, liver, and testis. The different levels of the enrichment are shown with varying degrees of grey colors. The high levels of the enrichment at ECR11 and 18 are evident with darker-color vertical lines, the positions of which are indicated with *. The lower panel represents the genomic structure of the Peg3 domain: paternally and maternally expressed genes are marked with blue and red colors, respectively.

According to earlier comparative genomic analyses [7,14], the 500-kb genomic region of the Peg3 domain can be divided into three regions: the middle 250-kb genomic interval harboring no obvious ORFs and the two small flanking regions containing all of the 7 imprinted genes (Figure **1**). Although no obvious ORFs have been identified from the middle 250-kb region, comparison of three genome sequences, including human, mouse and cow, immediately revealed that this interval contains more than 20 small fragments with high levels of sequence identity among the three species. Interestingly, the relative order, spacing and orientation of these small regions are also well conserved among the three species. For more detailed analyses, we have selected 18 regions as Evolutionarily Conserved Regions (ECRs) with the two following criteria: greater than 75% sequence identity among the three species and greater than 50 bp in length. These ECRs have been named serially based on their relative positions to the bi-directional promoter for Peg3/Usp29: the closest, ECR1, is located 18-kb upstream whereas the farthest, ECR18, is 200-kb upstream of Peg3. The average length of the ECRs is 100 bp, ranging from 60 to 600 bp in length. The average sequence identity between different species is 80%, ranging from 75 to 90%. Our initial characterization of these 18 ECRs was further confirmed through inspecting additional genome sequences that have been derived from other mammalian species, such as rabbit, dog and cat (Mammal Conservation graph in Figure **1**). The Peg3 domains of these species also have all the ECRs similarly as seen in the three initial species. More detailed information regarding the positions and sequences of these 18 ECRs are presented in Material **S1**.

**Figure 2:**
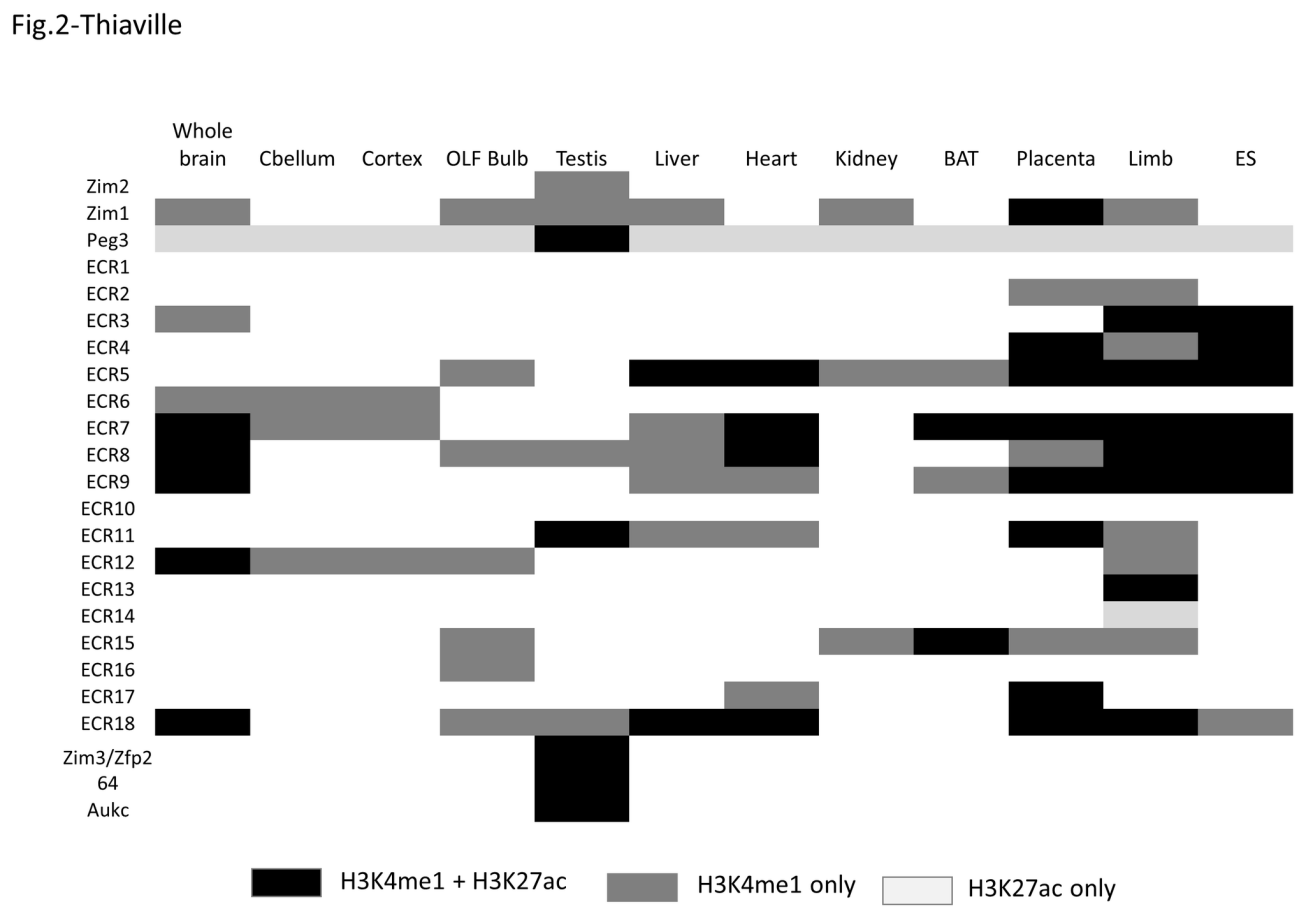
Summary of histone modification patterns on ECRs. The table summarizes the histone modification patterns of H3K4me1 and H3K27ac in the Peg3 domain. The histone modification status of each locus (the name on the Y-axis) was presented for each tissue (the name on the X-axis). Black rectangles indicate the regions with both H3K4me1 and H3K27ac modifications; the darker grey ones indicate the regions with H3K4me1 only; and finally the lighter grey ones indicate the regions with H3K27ac only.

The identified ECRs were also examined in terms of their histone modifications using the ChIP-seq data set of the ENCODE project [23]. Detailed inspection of the histone modification patterns revealed the following conclusions. The majority of the ECRs are closely associated with two histone modifications, H3K4me1 and H3K27ac (Figures **1** & **2**). According to recent studies, poised/inactive enhancers tend to be marked only with the H3K4me1 modification whereas active enhancers are either with both H3K4me1 and H3K27ac or only with the H3K27ac modification [22]. Out of the 18 ECRs, the positions of 16 ECRs, all but ECR1 and 10, coincide perfectly with those of the H3K4me1-enrichment peaks that have been derived from the various tissues of mouse (dark grey rectangles in Figure **2**). This indicates that the majority of ECRs are likely poised enhancers. Some of these 16 ECRs are also modified with the H3K27ac mark based on the overlap observed between their genomic locations and the positions of the H3K27ac-enrichment peaks (black rectangles). In particular, 5 regions (ECR 5, 7, 8, 9, 18) are quite often detected with the two histone modifications in several tissues, such as brain, placenta and embryo limb (Figure **2**), where the Peg3 domain is highly expressed. This suggests that these 5 ECRs are most likely active enhancers for transcription of the Peg3 domain in these tissues. In terms of the enrichment levels, ECR18 shows the highest levels for both H3K4me1 and H3K27ac modifications in these tissues, suggesting its dominant role in transcription of the Peg3 domain. In contrast to these ECRs, other ECRs tend to show somewhat tissue-specific modification patterns. ECR11 shows the two modifications only in testis and placenta, yet enrichment levels of the two modifications in testis are the highest among all the ECRs (also indicated by * in Figure **1**), suggesting that ECR11 may be a testis-specific enhancer for the transcription of the Peg3 domain. In sum, the majority of ECRs could be putative enhancers for the transcription of the Peg3 domain, and some of these putative enhancers, such as ECR11 and 18, might play major roles in the transcription of the Peg3 domain based on their histone modification profiles.

### Interaction of ECRs with nearby imprinted genes

**Figure 3:**
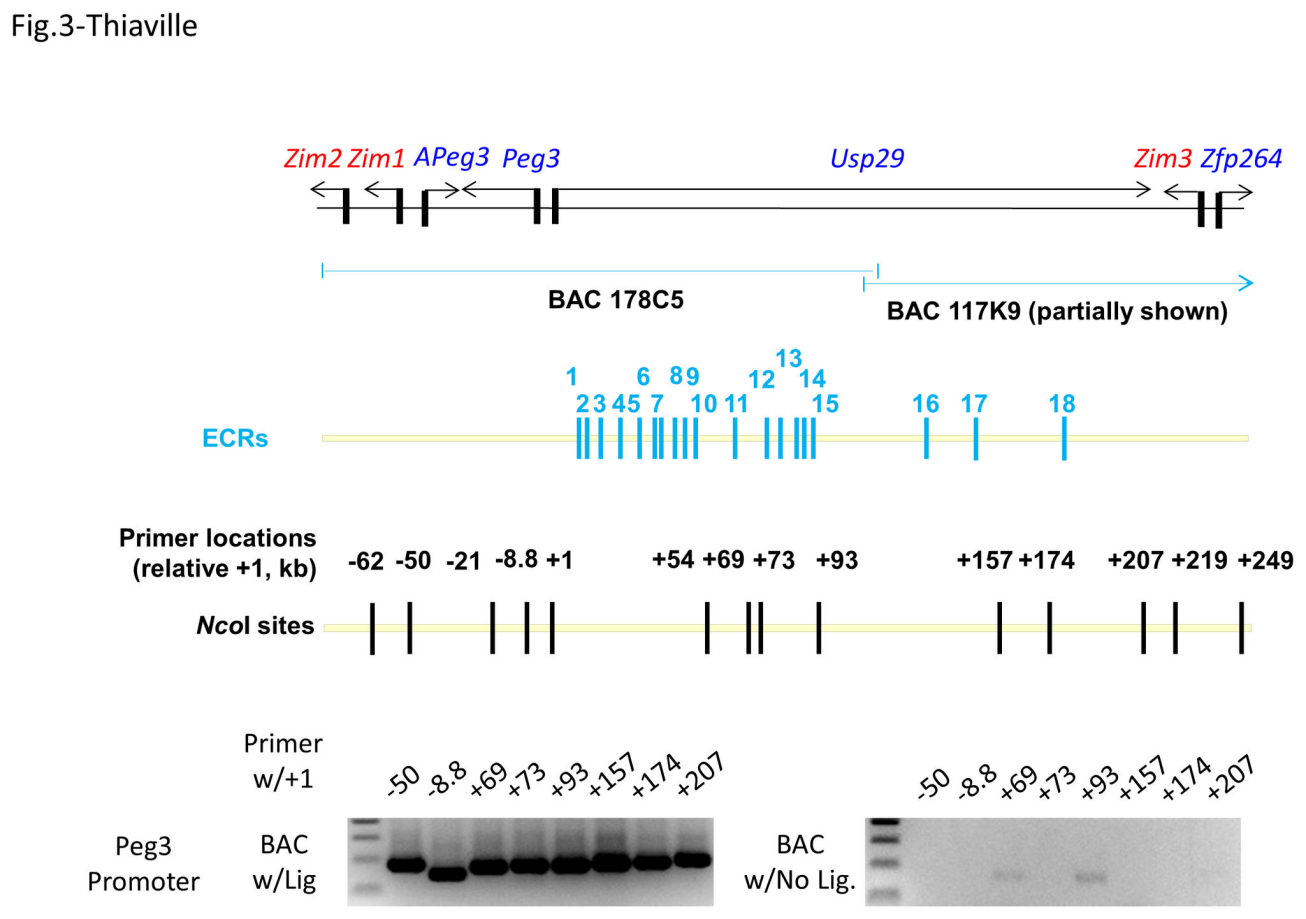
3C strategy for the Peg3 domain. The upper diagram details the genomic region covered by the two BAC clones (178C5 and 117K9) that have been used for preparing two control libraries, the relative positions of 18 ECRs, the relative positions of oligonucleotides that have been used for 3C experiments. Each oligonucleotide has been named with a numeric value to indicate its relative position to the transcription start site of Peg3. The gel images on the bottom panel show the results derived from the two sets of control experiments testing the efficiency and compatibility of each primer set. For both control experiments, the +1 oligonucleotide was used as a base primer, which intends to detect long-range interaction between the Peg3 promoter and other regions.

To further characterize the predicted functions of the ECRs, we performed the Chromatin Conformation Capture (3C) approach with the tissues derived from mouse [24–26]. First, *Nco*I was selected as the restriction enzyme for this technique since its recognition site (CCATGG) is located evenly throughout the Peg3 domain, and also closely associated with the promoters of most of the imprinted genes except Zim1 (Figure **3**). The two *Nco*I sites around the promoter of Zim1 are located within repeat elements, thus could not be used for 3C. Second, we designed more than 30 oligonucleotides that are located approximately 100-bp downstream of all the *Nco*I sites with the orientations of these oligonucleotides in one direction relative to that of the Peg3 domain. Each oligonucleotide is annotated with a numeric value to indicate its genomic position relative to the transcription start site of Peg3. Finally, we have obtained two BAC clones with a minimal overlap to cover the entire Peg3 domain (BAC 178C5 and 117K9). The DNA from these BAC clones were isolated, digested with *Nco*I, and subsequently used for preparing two control libraries for 3C experiments: one library was prepared with the ligation reaction step and the other was without the ligation reaction step (BAC w/Lig and w/No Lig. in Figure **3**). These two libraries were used as positive and negative controls for the PCR step in 3C to test the efficiency and compatibility of any given combination of oligonucleotide primers.

**Figure 4:**
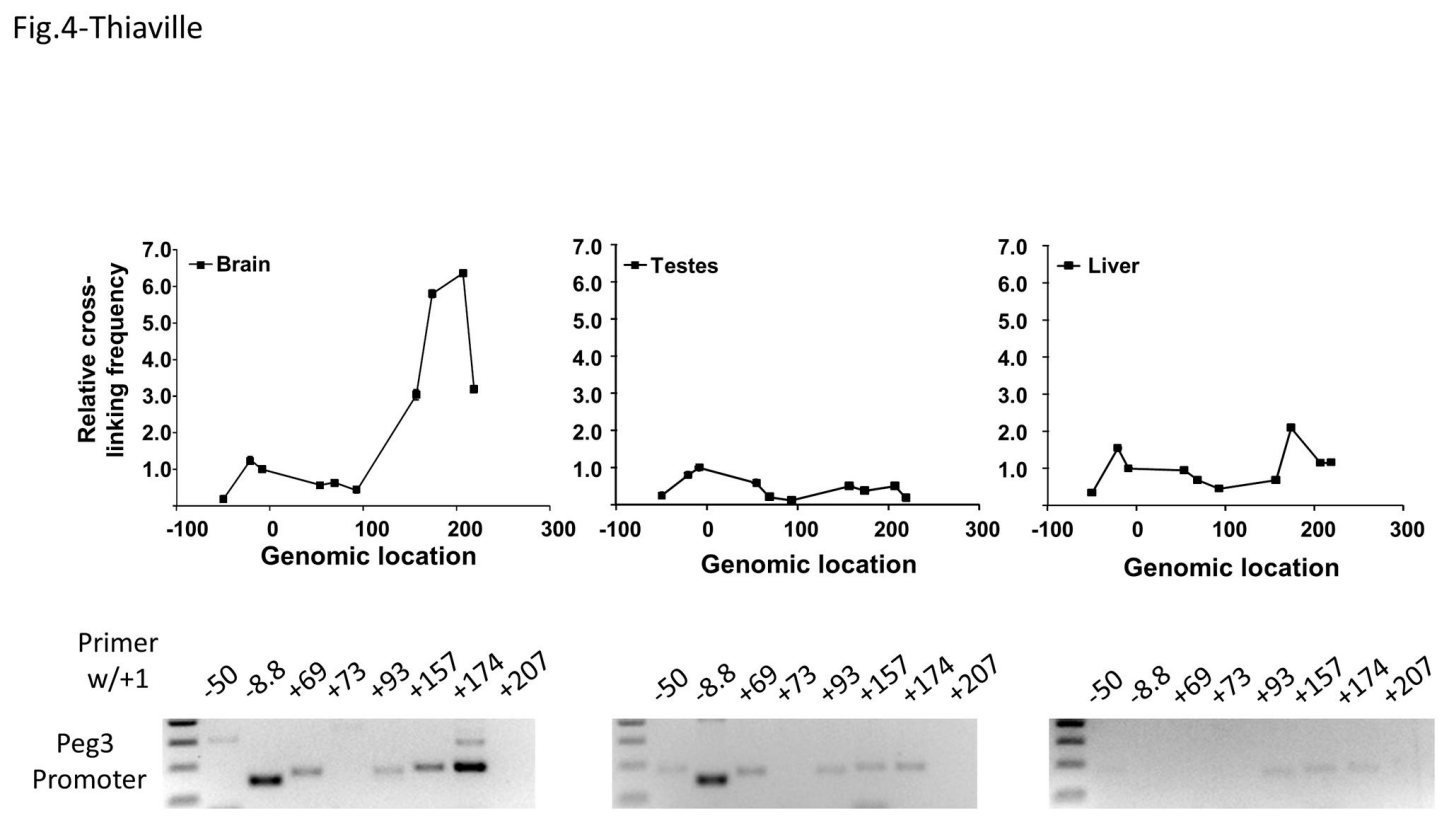
Interaction of the promoter of Peg3/Usp29 with ECRs. Potential long-range interaction of the Peg3 promoter was tested using the +1 oligonucleotide as a base primer along with a set of oligonucleotides that are derived from multiple regions of the Peg3 domain. The initial survey was performed with a fixed number of cycles (36 cycles) using the three libraries derived from neonatal brain, testis and liver, and these results are shown on bottom. Quantitative PCR analyses were also performed. The results are summarized with graphs on top. For this series of analyses, the Ct (threshold cycle) value for each primer set was first calculated from the control library with ligation, and subsequently used as an internal control for the normalization of the Ct values derived from the three tissue libraries.

For actual 3C experiments, three mouse tissues, brain, testis and liver, were harvested from a 10-day-old male mouse, and subsequently used for preparing 3C libraries. With the prepared libraries, we first tested potential interaction of the Peg3 promoter with the genomic regions containing the ECRs. After the feasibility and efficiency tests with the two control libraries (Figure **3**), a set of oligonucleotides were selected to be used as a paired primer individually with a base primer (+1 oligonucleotide). According to the results from the initial round of PCRs with a fixed number of cycles, two combinations of primers showed relatively high levels of the enrichment in two libraries: the -8.8/+1 pair in the brain and testis libraries and the +174/+1 pair only in the brain library (Figure **4**). The enrichment by the -8.8/+1 pair is likely over-represented in the two libraries due to the apparent close proximity between the two primers. The over-representation might have been caused by self-ligation of a linear DNA fragment spanning -8.8 through +1. Thus, the observed enrichment levels have been properly normalized in subsequent qPCR-based analyses. In contrast, the enrichment by the +174/+1 pair was consistently detected in the brain library with multiple trials, and also very significant based on the results of qPCR analyses, thus most likely representing the genuine enrichment of this genomic context in the brain library. Overall, this set of 3C experiments, using the +1 oligonucleotide as a base primer, concludes that the promoter of Peg3 most likely interacts with its 174-kb upstream genomic region in mouse neonatal brain.

**Figure 5:**
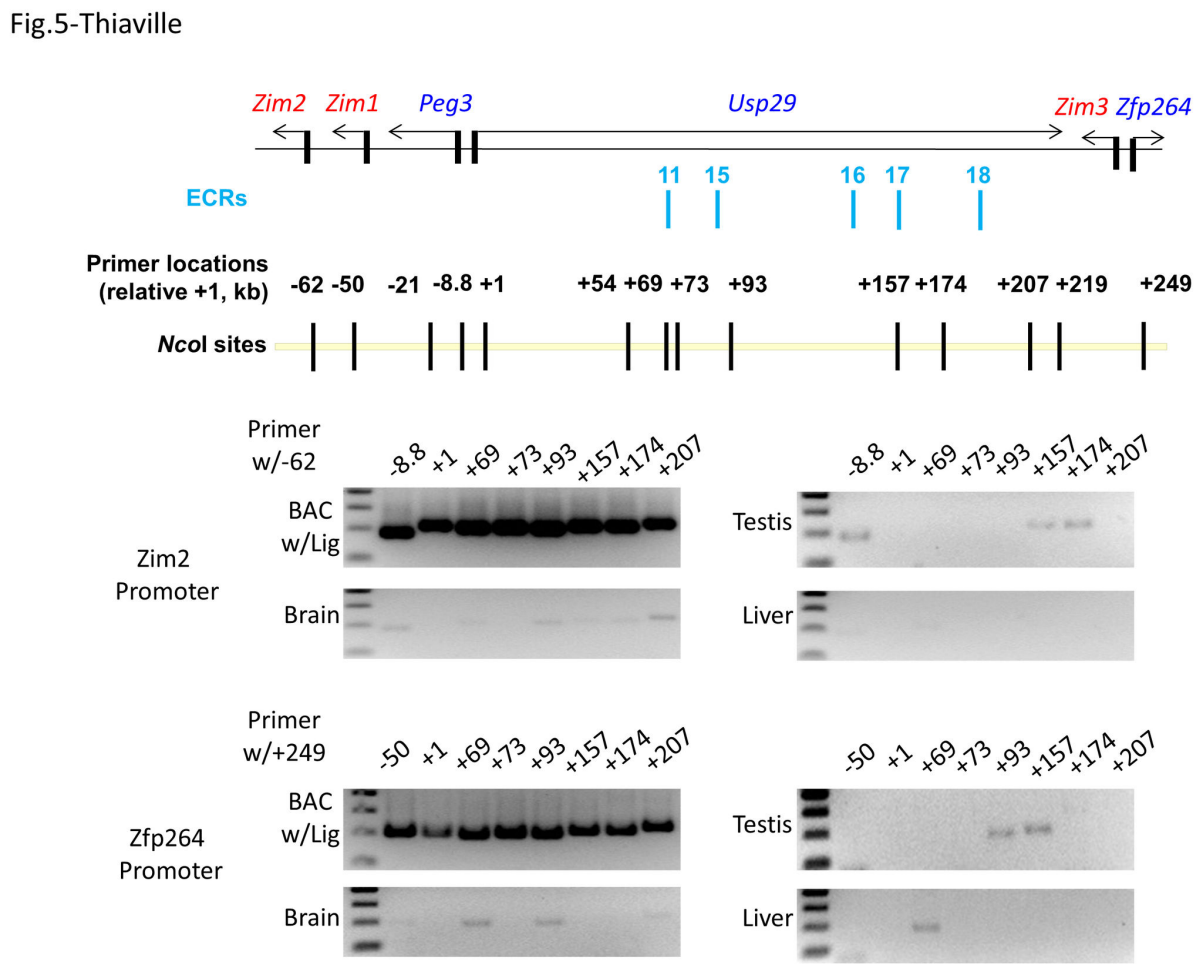
Interaction of the promoters of Zim2 and Zfp264/Zim3 with ECRs. Potential long-range interactions between the promoters of Zim2 and Zfp264/Zim3 versus other genomic regions harboring ECRs were tested using two oligonucleotides as a base primer (-62 for Zim2 and +249 for Zfp264/Zim3). The results are shown on the middle (Zim2) and bottom (Zfp264/Zim3) panels.

Similar approaches were repeated with two additional oligonucleotides, -62 and +249, as base primers to identify the genomic regions that interact with the promoters of Zim2 and Zim3/Zfp264, respectively (Figure **5**). The results from the 3C trial using the -62 oligonucleotide as a base primer indicate that both primer pairs (+157/-62 and +174/-62) show relatively low but consistent levels of enrichment in the testis library, suggesting that the promoter of Zim2 may interact with the 157-kb through 174-kb upstream region of Peg3. On the other hand, the results from another 3C trial using the +249 oligonucleotide as a base primer revealed that two genomic regions, the 93-kb through 157-kb upstream region in testis and the 69-kb upstream region in liver, might interact with the bi-directional promoter of Zim3/Zfp264. Although low levels of enrichment were also detected in the brain library, we conclude the enrichment to be marginal as compared to the enrichment seen in the mouse liver and testis. We also confirmed the identity of the majority of PCR products amplified during 3C experiments through cloning and sequencing. Collectively, the series of 3C experiments described above suggest that the promoter of Peg3 likely interacts with one prominent region (the 174-kb upstream region) in brain. The promoters of Zim2 and Zim3/Zfp264 might also interact with a similar region (the 157-kb through 174-kb upstream region) in testis. The observed long-range interaction is predicted to be driven by unknown *cis*-regulatory elements within this interval, such as ECR16-18. We further predict that ECR18 is the most likely candidate based on its proximity to the position of the +174 oligonucleotide and its dominant histone modification profiles (Figures **1** & **2**).

### Histone modification and DNA methylation of ECR18

**Figure 6:**
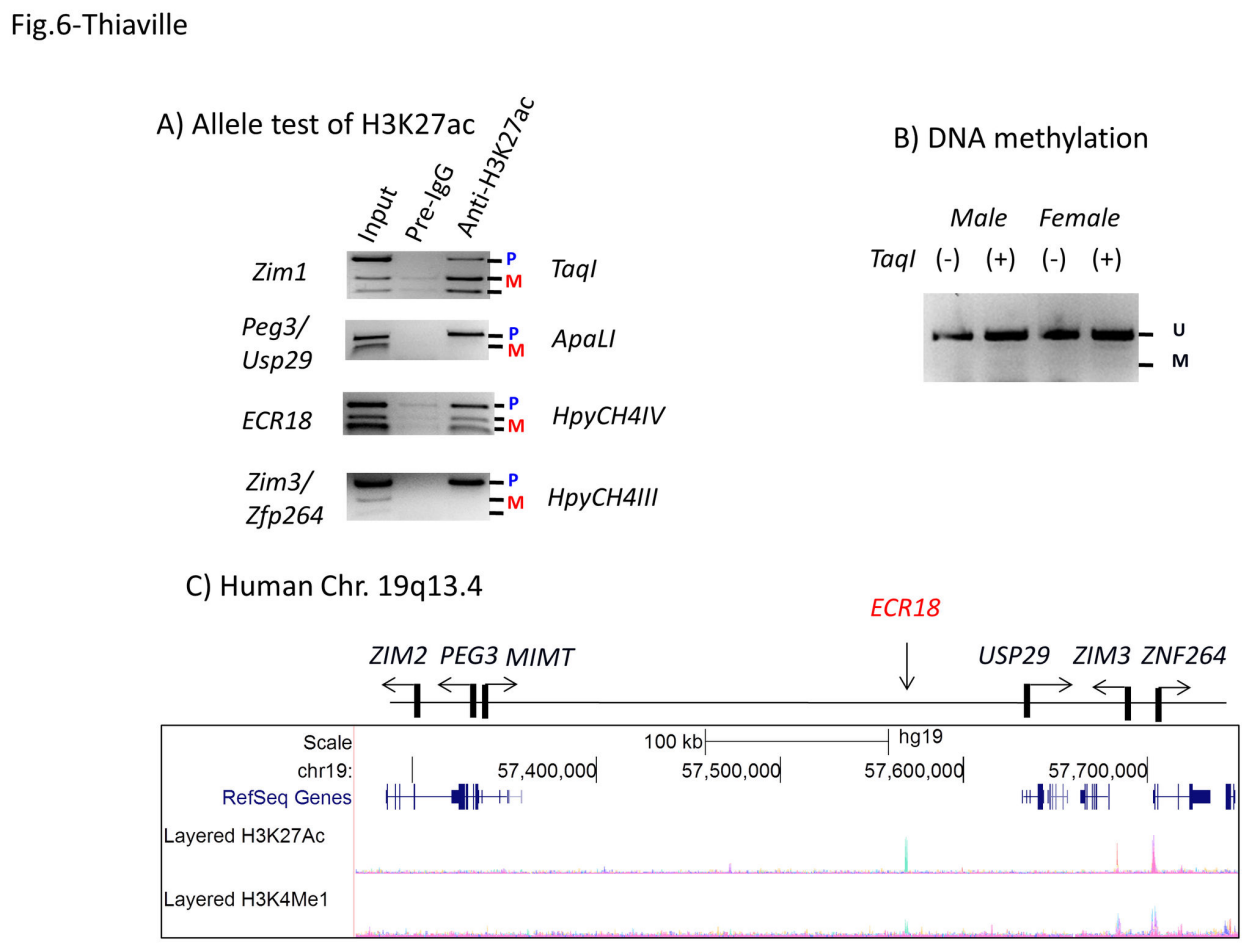
Epigenetic modifications and evolutionary conservation of ECR18. (**A**) Immunoprecipitated DNA with anti-H3K27ac antibodies was first amplified with PCR, and the subsequent PCR products were digested with an enzyme to differentiate parental alleles for a given locus. The promoter regions of imprinted genes show mono-allelic or one allele-biased patterns whereas ECR18 shows a bi-allelic pattern similar to those seen the input DNA. (**B**) DNA mthylation analysis on ECR18. The bisulfite-converted DNA from male and female neonates were used for PCR amplification. The amplified PCR products were digested with *Taq*I, and the digestion by this enzyme indicates methylation on the original DNA. (**C**) The panel represents a snapshot of UCSC Genome Browser showing the peaks of the two histone modifications, H3K27ac and H3K4me1, detected in the human PEG3 domain.

To further follow up the prediction described above, we decided to characterize several aspects of ECR18. First, we tested the allele specificity of the H3K27ac enrichment on ECR18 using hybrid offspring derived from the interspecies crossing of 129/B6 and PWD/PhJ [15]. We performed ChIP experiments with anti-H3K27ac antibody using the brain extracts prepared from the neonatal brain of the F1 hybrid. As expected, high levels of enrichment were detected in both the promoter of Peg3 and ECR18, but relatively low levels of enrichment were observed in the promoters of Zim1 and Zim3/Zfp264 (Figure **6**). Each amplified DNA was digested with an appropriate enzyme that can differentiate two alleles. As shown in Figure **6A**, promoters of the imprinted genes show the expected outcomes: the paternal-specific enrichment of H3K27ac in the promoters of Peg3 and Zfp264 and maternal-specific enrichment in the promoter of Zim1. On the other hand, similar tests clearly indicate bi-allelic enrichment in ECR18, suggesting that both alleles of ECR18 are marked with the H3K27ac modification. This further suggests that ECR18 is likely functional on both alleles.

We also tested the DNA methylation status of ECR18 since the sequence of ECR contains many CpG dinucleotides although the overall density of CpG is not high enough to be recognized as a CpG island (Material **S1**). As shown in Figure **6B**, the bisulfite-treated DNA that had been isolated from the brains of male and female neonates was analyzed with COBRA (Combined Bisulfie Restriction Analysis) [27]. The amplified PCR products were not digested at all with *Taq*I, the digestion by which is an indication for DNA methylation on the original DNA. These results confirm the unmethylated status of ECR18 on both alleles. This is unusual given the global DNA methylation on mammalian genomes other than the CpG-rich promoter regions of individual genes. This might be an indication for the active involvement of ECR18 in transcription of the Peg3 domain. Finally, we also examined the histone modification profiles of the human PEG3 domain (Figure **6C**). Human ECR18 also appears to have the two modifications, H3K4me1 and H3K27ac, yet the enrichment levels appear the highest among all peaks in the PEG3 domain. Again, this supports the idea that human ECR18 is an active enhancer for the PEG3 domain. Overall, these results clearly indicate that ECR18 is likely an evolutionarily conserved regulatory element for the mammalian Peg3 domain.

### Transcriptional activity of ECR18

**Figure 7:**
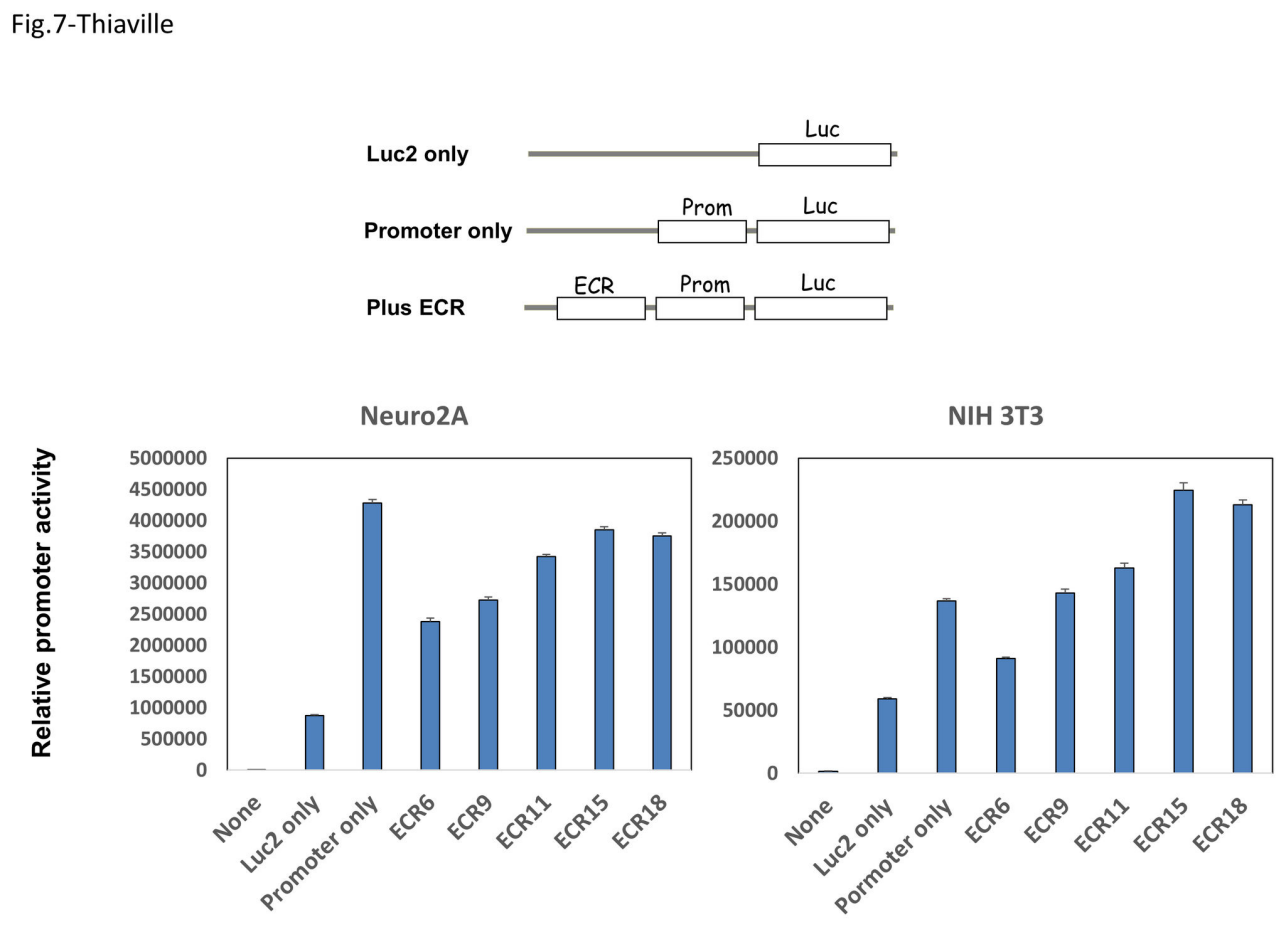
Transcriptional activity of ECR18. The diagram on top shows schematic representations of the reporter constructs that have been used for the current study. The graphs on bottom summarize the results derived from a series of reporter assay with these constructs. These assays were conducted in triplicates in Neuro2a and NIH3T3 cells and normalized with the β-Gal activity of an independent reporter construct. This series of reporter assays were repeated three independent times.

Several ECRs, including ECR18, were also tested for their predicted role as an enhancer in the transcription of the Peg3 domain. For this series of analyses, we first modified an available reporter system with the Luciferase gene into a promoterless reporter system, exhibiting very minimal transcriptional activity (Luc2 only in Figure **7**). Then, we cloned the two bi-directional promoters of Peg3/Usp29 and Zim3/Zfp264 into the immediate upstream region of the promoterless reporter (Promoter only). Although we initially tried both promoter regions, we were able to detect boosted transcriptional activity only from the ‘Promoter only’ construct with the bi-directional promoter of Peg3/Usp29. Thus, the reporter construct with the Peg3/Usp29 promoter was used for testing the enhancer activity of ECRs (plus ECR). A series of the constructs with different ECRs were individually transfected into Neuro2a, NIH3T3, HeLa and HEK293 cells, and their transcriptional activities were measured in multiple trials. The majority of ECRs tested in this series of analyses appeared to be repressors in Neuro2a, and HeLa and HEK293 cells (Figure **7**). In contrast, ECR15 and ECR18 are somewhat different in NIH3T3 cells: both ECRs boosted the transcriptional activity of the Peg3/Usp29 promoter as a potential enhancer (Figure **7**). Overall, these results confirm that the majority of ECRs, including ECR18, are involved in the transcriptional regulation of the Peg3 domain as *cis*-regulatory elements.

## Discussion

In the current study, we performed a series of analyses to characterize potential roles of 18 ECRs in the Peg3 domain. The majority of these ECRs are potential *cis*-regulatory elements based on their association with two histone modifications, H3K4me1 and K3K27ac. The results from 3C also identify one genomic region harboring ECR18 as a main site physically interacting with the promoters of Peg3 and Zim2. Detailed analyses further demonstrate ECR18 as a putative enhancer or repressor for the Peg3 promoter. Overall, these results suggest that ECR18 likely plays key roles as a distant *cis*-regulatory element for the transcription and imprinting of the Peg3 domain.

The mammalian Peg3 domain harbors 18 evolutionarily conserved regions (ECRs) with unknown function (Figure **1**). According to histone modification profiles, these ECRs are potential *cis*-regulatory elements for the Peg3 domain (Figures **1** & **2**). The histone modification profiles also provide several hints regarding their roles as *cis*-regulatory elements. First, the functions of these ECRs are predicted to be tissue-specific, since their histone modifications are limited to a subset of tissues or specialized cell types, such as ECR11 in testis and placenta, ECR15 in brown adipose tissue (BAT), and ECR17 in placenta. By contrast, the histone modification patterns of other ECRs are more ubiquitous, such as ECR5, 7, 8, 9, and 18 in brain, liver, placenta and embryonic limb. Second, each ECR’s contribution to transcription and imprinting might be variable based on the different levels of histone modifications (Figure **1**). In particular, the modification levels of two ECRs, ECR11 and 18, are much higher than those from the other ECRs. Since ECR11 is also tissue-specific, the functional contribution by this ECR is likely very significant in a subset of tissues, such as testis and placenta. On the other hand, ECR18 displays the highest levels of enrichment in the various tissues where Peg3 and Zim1 are highly expressed, in particular brain, placenta and embryonic limb. Thus, ECR18 is predicted to be the major ECR contributing to the transcription and imprinting control of the Peg3 domain.

The ECRs of the Peg3 domain are very unique. First, the number (at least 18) of and the relative genomic size (250 kb in length) occupied by the ECRs are unusually high and large given the overall size of the Peg3 domain (about 500 kb in length). This relatively large number of potential *cis*-regulatory elements might be designed for multiple genes within the Peg3 domain, not just for a single gene. This idea is further supported by the results from 3C experiments that the ECRs interact with the promoters of several genes in the Peg3 domain, including Peg3, Zim2 and Zfp264 (Figures **3**, **4**, and **5**). Second, the order, orientation and spacing of 18 ECRs within the 250-kb genomic interval has been very well preserved during mammalian evolution, indicating the presence of unusual constraints on maintaining the overall genomic structure. This constrain might be related to the exon/intron structure of Usp29 in the case of the mouse since the entire genomic interval is part of Usp29. However, the observed constraints might be more related to potential regulatory modes, by which the ECRs control the surrounding genes. It is well known that the genes in each imprinted domain are co-regulated through shared *cis*-regulatory elements [1–3]. Thus, we predict that some of the ECRs might also function as shared *cis*-regulatory elements for the co-regulation of the Peg3 domain. One likely candidate would be ECR18 since this region appears to interact with both Peg3 and Zim2 according to 3C experiments (Figures **4** & **5**). Taken together, these data suggest that sharing *cis*-regulatory elements between the imprinted genes might have been a major driving force for maintaining the overall genomic layout of the 250-kb genomic interval.

**Figure 8:**
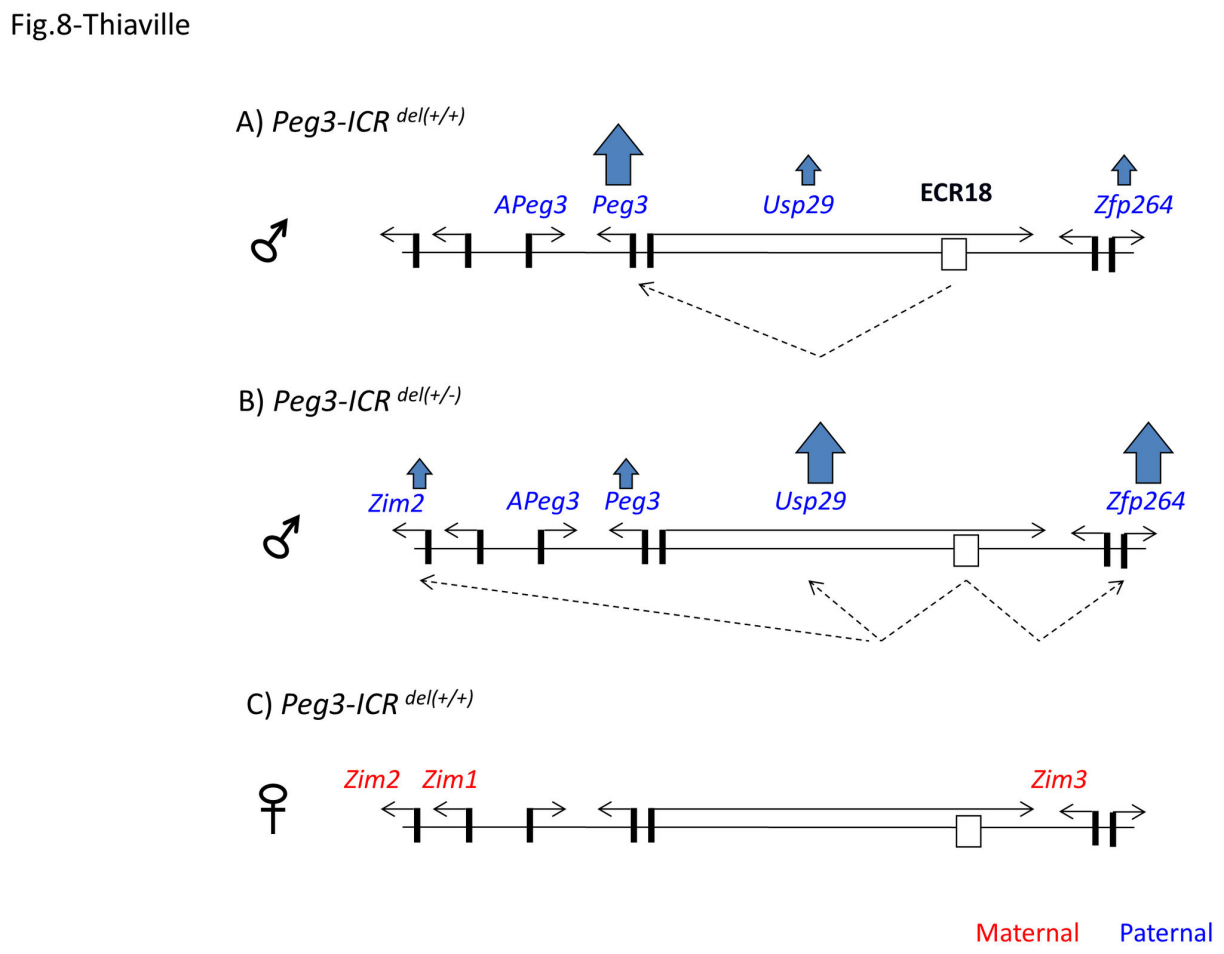
ECR18 as a shared enhancer for the Peg3 domain. (**A**) The diagram represents the paternal allele of the Peg3 domain with each gene being indicated with a horizontal arrow. The transcriptional level of each gene is also indicated with a vertical arrow: the thicker or thinner arrow indicates higher or lower expression levels for a given gene. Potential interaction between ECR18 and the promoter of each gene is indicated by a dotted line. (**B**) The diagram represents the paternal allele of Peg3 domain in the mutant animals that have a deletion in the Peg3-DMR, an ICR for the Peg3 domain. (**C**) The diagram represents the maternal allele of the Peg3 domain in the wild-type animals. The maternal allele in the mutant animals is not shown since the mutational effects are very minimal.

According to previous studies [15], the transcription and imprinting of the Peg3 domain are regulated through one ICR, the Peg3-DMR, a 4-kb genomic region surrounding the 1^st^ exons of paternally expressed Peg3 and Usp29 (Figure **8**). Deletion of this ICR on the paternal allele resulted in dramatic down-regulation of Peg3 but up-regulation of the neighboring genes, including Usp29, Zim1, Zim2 and Zfp264. The same mutation also caused changes in the imprinting status of Zim2, from maternal-specific to bi-allelic expression. On the other hand, the mutational effects on the maternal allele are very limited [15], and thus will not be discussed hereafter. The observed mutational effects on the paternal allele could be explained with the following model. Transcription of the Peg3 domain might be driven by one shared enhancer. Peg3 is likely the main user of this enhancer and out-competes the other imprinted genes in the domain. ECR18 may be this shared enhancer given the results from the current study. If this is the case, ECR18 might exert its predicted roles in the following manner. For the transcription of the Peg3 domain, the interaction of ECR18 with Peg3 might be dominant. Thus, deletion of this dominant user in the mutant mice might allow access of the ECR18 to the other imprinted genes, resulting in down-regulation for Peg3 but up-regulation for the other genes. A similar logic could also be used for explaining the changes in the imprinting status of Zim2 in the mutant mice: Zim2’s accessibility to ECR18 on the paternal allele might be responsible for converting the normally silent to active state, resulting in the expression of both paternal and maternal alleles of Zim2 (Figure **8**). Given all the available data obtained so far, this is the most likely model explaining the transcription and imprinting of the Peg3 domain. Thus, testing this model through mouse genetics should be of great interest in the near future. Also, potential mutagenesis experiments targeting ECR18 will further characterize the *in vivo* functions of this unknown enhancer.

## Materials and Methods

### Ethics Statement

All the experiments related to mice were performed in accordance with National Institutes of Health guidelines for care and use of animals, and also approved by the Louisiana State University Institutional Animal Care and Use Committee (IACUC), protocol 10-071.

#### 3C (Chromatin Conformation Capture)

The 3C method was performed as detailed in the Current Protocols in Molecular Biology Handbook unit 21.11 in Supplement 74 [26]. In brief, the two mouse BAC (bacterial artificial chromosome) clones, RP23-178C5 (Invitrogen) and RP23-117K9 (CHORI), were used for generating the two control template libraries. These BACs cover the majority of the Peg3 domain (nucleotide positions 6,610,343-6,929,458 in mouse chromosome 7) with an approximately 9,000 bp overlap (6,793,948-6,802,969). The 9-kb overlap region was not used for designing oligonucleotides for 3C analysis. The purified DNA from these two BACs (total 10 µg with an equal ratio) was digested with *Nco*I, religated, and finally prepared for the control template libraries. For actual 3C experiments, the three tissues, brain, liver and testis, were harvested from a 10-day-old-male mice. Each tissue was weighed, homogenized in PBS, crosslinked with 1% formaldehyde for 10 minutes (mins), and finally divided into several fractions at the concentration of 10 mg per aliquot. Each aliquot was used for *Nco*I digestion, religation, and DNA purification. A separate aliquot was also treated similarly but without religation, and the purified DNA from this treatment was used as a negative control.

For PCR analysis, each primer was designed from the region that is 80 to 150-bp downstream to a given restriction site of *Nco*I (Figure **3**). The efficiency and compatibility of a given primer set were first tested using serial dilutions of the control template libraries that had been prepared from the two BAC clones. For an initial survey, a fixed number of PCR (36 cycles) was run using each base primer along with a panel of paired primers (Figures **3**-**5**). For quantitative PCR (qPCR) analysis, 1 µl of either the control or the 3C libraries from mouse tissues was used as a template with SYBR Green Premix reagents (BioRad). The parameters for PCR are as follows: 95°C for 4 mins, 40 repetitions of the following cycle of 95°C for 15 sec, 65°C for 30 sec, 72°C for 30 sec. A camera capture setting was included after the 65°C step to monitor the formation of PCR product. A melt curve step ranging from 55–95°C with a hold of 10 sec and a temperature increment of 0.5°C was included at the end of the PCR to monitor the quality of PCR product.

### ChIP (Chromatin Immunoprecipitation)

For ChIP analyses, mouse brain was harvested from 1-day-old F1 hybrid that had been obtained from the interspecies crossing of 129/B6 and PWD/PhJ [15]. The harvested brain was homogenized, crosslinked with 1% formaldehyde for 10 mins, and fractioned into several aliquots (5 to 10 fractions per brain). Each aliquot was used for a given ChIP experiment. The immunoprecipitated DNA from each ChIP was dissolved in 50 µl of TE (pH 8.0) for further analysis. The current study used the following antibodies: the polyclonal antibodies against H3K27ac (Abcam, Cat# ab4729). For allele tests, each PCR product from ChIP DNA was digested with restriction enzymes that can differentiate two parental alleles. The detailed information regarding the sequences and positions of the primers that have been used for this study is available (Material **S1**).

### DNA methylation analysis

DNA was first isolated from the brains of male and female neonates, and these DNA were treated with the bisulfite conversion protocol [27]. The converted DNA was used for PCR amplification using the two following primers: ECR18-bis-a (5’-GGGGTTTTTTAGAATTTGTTTTATGGAGGT-3’) and ECR18-bis-b (5’-CTCTATCTCTTTAAATATATCCAAAACTATC-3’). The amplified product was digested with *Taq*I and separated on 2% agarose gels to survey the CpG methylation status of the original DNA.

### Reporter assay

For promoter assays, we first modified the promoterless β-Geo reporter [18] by replacing the coding region of β-Geo with that of the *luc2* gene. The 1-kb genomic region containing the bi-directional promoter of Peg3/Usp29 was cloned into the upstream region of the promoterless vector, and finally several ECRs was individually cloned into the 5’-side of the Peg3/Usp29 promoter. For the luciferase assay, Neuro2a, NIH3T3, HeLa and HEK293 cells were plated in 24-well plates (0.75 x 10^5^ per well) with the DMEM plus GlutaMAX medium containing 5% fetal bovine serum and 1% antibiotic-antimycotic (GibcoBRL). On the following day, the cells were transfected with 1 µg of each reporter construct using 2.5 µL Lipofectamine 2000 (Invitrogen). Fresh complete media was added 6 hrs post transfection, and total cell lysates were harvested in 100 µL of 1X reporter lysis buffer 48 hrs post transfection (Promega). The luciferase assay was performed for each promoter construct in triplicate according to the company’s protocol (Promega). We also performed a similar set of transfection experiments using an independent β-Geo reporter construct to monitor the transfection efficiency. Thus, the initial values from the luciferase assay were normalized with the β-Gal activity.

## Supporting Information

Material S1 **Sequence information for 18 ECRs and oligonucleotides used for 3C and ChIP analyses.**
(DOCX)
