# Supplementary material for: Identification of an Evolutionarily Conserved Cis-Regulatory Element Controlling the Peg3 Imprinted Domain
Source: PLoS One. 2013 Sep 10;8(9):e75417. doi: 10.1371/journal.pone.0075417 (PMC3769284; doi:10.1371/journal.pone.0075417)
Supplement: Material S1 — Sequence information for 18 ECRs and oligonucleotides used for 3C and ChIP analyses. (DOCX) [file pone.0075417.s001.docx]

1. The mouse sequences of 18 ECRs are shown below. The regions in uppercase represent oligonucleotides for PCR amplification.

>ECR1([chr7:6700859+6701125](http://genome.ucsc.edu/cgi-bin/hgTracks?hgsid=309458049&db=mm9&position=chr7:6700859-6701125&hgPcrResult=pack)) 267bp

AGAGAGGTGAGCCTTGAAAGGTtactgtcttttattctttttttttttaa tccccctccaggttacaagttagtgtggatggcaggtctcggaatgaatt ccaaatctgccagatgcctgaagtcttcaataaccactgggtacatttct aggctattaaatttgaaagccataatgaaatgtatcattctggaaaggta gattctaagaacttggcaatgtcaaaggaatgccttcatcaaaatGTGCT

CCAAGCCCTTGTCAGCT

>ECR2([chr7:6701804+6702141](http://genome.ucsc.edu/cgi-bin/hgTracks?hgsid=309458189&db=mm9&position=chr7:6701804-6702141&hgPcrResult=pack)) 338bp

CTGGCTGACCCCATTCTTAGGAttaaaatttaaaactcgaatttaaatgt caggggactattttaataagtatctccccaccccacccctcaaaagggag gaaaaggcatgcctggaaatggtgactttagtaggagcagaaacttcctt ccaactttcaaggaacagataactcatccgtcccacaaagcgtgccaagg cctagaaaccagtggagttcttcccagttcgttttaattaactggttcac agcggtttatgatccccgagtgattttaattggaaggaactcagcataat gaaaataacaacagctGACAGTTGCGCAATGTCCCCTC

> ECR3([chr7:6706165+6706435](http://genome.ucsc.edu/cgi-bin/hgTracks?hgsid=309458401&db=mm9&position=chr7:6706165-6706435&hgPcrResult=pack)) 271bp

CCCGAGAAGATGACGAGGACGGTatgctggtcagattcctctcccacctc ctacctcctcccagcctgtcgaagagctggaaaatttcaatcccttcctt tcccactccagctgcagtttccatggtaatagagtgaacctgactctgtg gctaacaacagctaacagctcttgaaagggtctaagccagaacaattgga gacaacctaaattggtgggggaagggagggctccctgaatccctgactaA CTCACTGTTAGCTTATGCAAG

>ECR4([chr7:6712479+6712740](http://genome.ucsc.edu/cgi-bin/hgTracks?hgsid=309458517&db=mm9&position=chr7:6712479-6712740&hgPcrResult=pack)) 262bp

TCGCCTCGGTGGACACGCTTCTGcaagccaaacaattgctttgctttgga tagccgggcccatcaaagatggactcagtctgataaacacgcctctgcat gccaaccaatccagcggggtctcactccagagaccacacttctgcagatg acgttaatgtcagtccttttctgtctttgaaagctggacagcacctgtcc tctgctgcccccagacccttcatagaacagtaccatgctTCAGAGAGACT

CCGGATTAGCCT

>ECR5([chr7:6722969+6723270](http://genome.ucsc.edu/cgi-bin/hgTracks?hgsid=311265999&db=mm9&position=chr7:6722969-6723270&hgPcrResult=pack)) 302bp

GGTTCTTGGGTGGCAGGCCCAAGgattcagctccccctccccttctgctg gggtcagggaaatgagccttcttcctggcccctgcggggcctccagccgg cagctggagggattaaggaaaagaggttcctggcgtccctgagtctggag tggctgcccaggatgtcccaggagaggcgagtgggccagagggctggagg ctcccaaatcaatcagaggtctgaaggtcactgaatggcgacctttgaat caggatcccggtaaccctgaggctttcgcaCACTCCGAGGGGGTGGAGCA

GA

>ECR6([chr7:6729136+6729374](http://genome.ucsc.edu/cgi-bin/hgTracks?hgsid=311266137&db=mm9&position=chr7:6729136-6729374&hgPcrResult=pack)) 239bp

TATGGTGTGGGATCCATCGTAGctattctaaggaaggcctgctggggtat tcagatggtgggatcctcctattcagccccatcgctgtgttttccctctt ctctctgatatttttggtggcacggcaaatattttctgcctgcctctttc tatgaaacactcagcaaaagtgggaggtgagccacagcttgtacttattt gggctttccaaacgcgTGGCACGGTTCTGTGTGCTTTGC

>ECR7([chr7:6729982+6730212](http://genome.ucsc.edu/cgi-bin/hgTracks?hgsid=310515571&db=mm9&position=chr7:6729982-6730212&hgPcrResult=pack)) 231bp

GGCTCCAGGAACTGGCTGTCAGggaggagagggtgtagctgtcctggggt gatggaatgtagccagcagctgtctcttctctatttgagcctgtacactg gcagggcactgtcacgcaaagcttccccataagagcatggggatagatga tcactctcaggtcatttatctgtgtgaggaaggccagcttcctttgcacc tagaagaatAGCCTTAGCTGGCCTGCTGTGG

>ECR8([chr7:6734275+6734643](http://genome.ucsc.edu/cgi-bin/hgTracks?hgsid=311266385&db=mm9&position=chr7:6734275-6734643&hgPcrResult=pack)) 369bp

GGGGTTAAGCCTTCTAGCCCAtttgtcgctggaaactcagtttgaatccc accctggaattttcctccgagctacgaagccaggcgagctgccaagtcat atatacattgccgggtagccaggcaactccgtgctttttctgggaaggaa cacaatgtggtcactgtttctgcatcgccaggttccagatgggagaaaga ctttaatccccagcttcaaggctgtttggcaaaactcactctggagcccc tgcctgggccggtgttcgtttgctgtgaccctccggtgctgccattttgt gacattctgtttctggtgtttaaaattcaatccttgcaaactagggagGT

AGGCTCTGTCAGTGACGCT

>ECR9([chr7:6737387+6737614](http://genome.ucsc.edu/cgi-bin/hgTracks?hgsid=311266591&db=mm9&position=chr7:6737387-6737614&hgPcrResult=pack)) 228bp

CTGGCTACACTGAATCCCACAGcttttgctgatagttttaaaccaaggac aaacagcacaaccaagacaagatcctatgtgtgcacgtgtgtgcaggcag ctgcatccaaggacatgattagccgttaagccccctctggatccatgcat cccttgttcagcacattctcttcccagcaaaaaggctggagccccaacgg tgttgtGTAATTCCCACCACACCCCAAG

>ECR10([chr7:6740801+6741262](http://genome.ucsc.edu/cgi-bin/hgTracks?hgsid=311266675&db=mm9&position=chr7:6740801-6741262&hgPcrResult=pack)) 462bp

CCTGAACATCACACATCTCCCAagtggaggggaatgaactccagtgaagg gtgggtggattggaaagttggccccaaacaagacaaaatagcttgatttt taacacatggcccagtttccagagacacactcaagctcatcaacccacca tgggatttttttccatcaggcagaaaagccaggttgattccaagaaatat atacattgccgcacaattaggcaaagggagagagtccttggagccttggg gccatgtggtcagtatttgggaggcatccacttcctgatggtaaatagat ttaagcacgccatctgttgtgtgcttatttcccaggacctgttttctttc tctaattttcagttctgacaaccttgttttctttttcttgacttaaaaaa aaaagtgacataaaccagtatttgagggtttttattttttCTCATGTGCT

AGGCCTGATGAC

>ECR11([chr7:6753381+6754001](http://genome.ucsc.edu/cgi-bin/hgTracks?hgsid=311266797&db=mm9&position=chr7:6753381-6754001&hgPcrResult=pack)) 621bp

GGTGGGGGAAGCAAAGCATCTCAgaacactagctgttaggatgtcagttt catcaggttgccaagtaaaaccccacagcaactgcccaccatggaatttt ccctgattaatgacctacgaatgcccggctggcgtccaagctctatatac attgacatccaactcaggaagggaaacaattttcccagaaacagcacaac atggccactattcttcagatgtccgcttctaaaaccataaaaatatttca tagtagccgtctgtttgtggaccatatccctgggtcgtggacataagtgg gttttctctggtgttctgggagggtggactgtcctcatctgtagatgact tgaatgcctgttcccagggcccttgcagcccatgtgccacactcttcctg ttcagtgttggcacagagcggtaacctacttgaagacgtgtggcttgaac tacattgtataaacattgaaataataagtcaaacaagcaagtacattttc actcttgtgcctctgtacccaactttataaatctccaaatgtgtgcattc aaaggagctgtgtgttctcccccaatagctgccttgtgtaacagtaaaCA CCATTATGCAAACATGACTGC

>ECR12([chr7:6763368+6763594](http://genome.ucsc.edu/cgi-bin/hgTracks?hgsid=311267093&db=mm9&position=chr7:6763368-6763594&hgPcrResult=pack)) 227bp

CCGGTGTATTCGTGTTCACAAGagagactgctgagcccatcccccagcca ggacacttggccctgagttgctcaagagccctcatcctcagtcattgcag ggacttgttgccatggaaactgcagccagtgtttccatggagaaagagct gcagagtctggaattttccaggttctaaaggtgaatgtatattctcttga aaacaACTCTGAATGCTTTCCCCCAGG

>ECR13([chr7:6767731+6768003](http://genome.ucsc.edu/cgi-bin/hgTracks?hgsid=311267187&db=mm9&position=chr7:6767731-6768003&hgPcrResult=pack)) 273bp

GGAATGCGCAAGTTTCTAATTGgaaacttgtgctcttcaaaaaggagtct ttgaactttgtttcctggcccccgtcgacagattgtacttagttcatgga ctcaccgtggttcatttagcttggctctcggcaacaaaccaagcaataca tacatctcctcccctgcaggaagcggttgggtttcttggacgtatctcaa ttggcctctgttgggaagacaagcatttccaaaccctaaaagcagtcagg aCTACCAGCTATATCCTTCCAAG

>ECR14([chr7:6773011+6773303](http://genome.ucsc.edu/cgi-bin/hgTracks?hgsid=311267257&db=mm9&position=chr7:6773011-6773303&hgPcrResult=pack)) 293bp

CATCTGGTAGTGTGGGGAAGACttgagcctaacctaaataaacagaaaag gaattttggtggtcgaaacctctctgacaggagactctcagtctttcaga aatagtttcaatgtcaacatctggtccaggtgccagataaaactttgctt caacaacccacctcagaattttttccctgattaatgagctcagtcgtgga gtgaaatccaagcagtatatacataccactgcccagctcaggaggagact ggctttctcggaaagagtacaACCCAGCCAGTGTTCTGGGTGC

>ECR15([chr7:6775357+6775685](http://genome.ucsc.edu/cgi-bin/hgTracks?hgsid=311267299&db=mm9&position=chr7:6775357-6775685&hgPcrResult=pack)) 329bp

GTCTGCTGTTTCTCTGTAAGGAatttggtcaacagctttttttttccctg aagaaagtgaaggatgtgaaatggtggggttgtgtagcagtagagacctc ctaagaagacgatggagccttggcttggctctgggaaggaaaaatgttct gacatttcactgatccagtggaaaatggaacgcccaacctctgttcagct ctgtctctgtgcgcctcattaattccctagaaagaacttctggaagtcaa attactaggtcacaggggatgaaaaggtctcatgacttgcttggattagt aaattttACTCCTTGTCAGGAGGTTCAGC

>ECR16([chr7:6822432+6822676](http://genome.ucsc.edu/cgi-bin/hgTracks?hgsid=311267405&db=mm9&position=chr7:6822432-6822676&hgPcrResult=pack)) 245bp

TCTATGCCCATCACCACTATCTctgagggagattttgcaactctgtggcc ttagcaacagaggcaggcccagaccatcaccatggaaactgcatcaggac tgcataaagatgggaggagctgggaactggaattttccattttttggatt cagttctaaaccatgactggcacacagtggtccttcattaaatgtttatt gagaaaacaaatacctgaaacctaACGCAGACAGCAGGGGCTGGA

>ECR17([chr7:6838133+6838559](http://genome.ucsc.edu/cgi-bin/hgTracks?hgsid=311267487&db=mm9&position=chr7:6838133-6838559&hgPcrResult=pack)) 427bp

GGCCCAAATGCCAGCCATGTTGagattgagcaaagccgtgctgtacaata ggaatttctcccacaagcatatcagaaaggaagtttggcatcatggtgtg gaaatgtgaaacgtgctcacactagagccagctgtgtactgagctcctca caaacacataggacaggaatgcattgtgtcagacaagggagagaaaaggc agccagccccaggaaattaaagaaaagctggctatcggctgcatctaggg acacggcgtgccgagcagacagctgcggtttcaatactctgctggccatg gggagctggttccaagaagctgctgaccccctgagtcctgtggaaattca gttacttcctggacctcaggtgaacctttagctcacctcccataggattc cacccaCAGTGAGTCCTGGGAGCCCGG

>ECR18([chr7:6866573+6866967](http://genome.ucsc.edu/cgi-bin/hgTracks?hgsid=310514715&db=mm9&position=chr7:6866573-6866967&hgPcrResult=pack))395bp

TGCTGCCTGCCTGCCAGCAGAAGccccgggcgggtcagaagcagtttgac ataccgcagcggccaacacagctgttgaggggggattagctgcagggaaa gccgatccagtgacaaatggtttaggtatgtccgcccctgggaaatgtga tctccacggtttgcactatagaaacttgtttgtttcaaaagatgaggaac atgtttgttgtctgtgtctccagagaaatcacaacgttgacagctctgga tatacccaaagagacagagtacaaaaaaaccaattacacgtaggctgcgc tcataatcaacaaatgctggtttgggaattgctgtgtgaagattcctctc ggcagcctgctcagaatgtacgTCTGGTGACTTCTGAGATGTCAC

2. The oligonucleotide sequences for 3C experiments are shown below.

> -62

AAAGGTTGGAAGACACTCACAGATGAG

> -50

CCTAATCACATGGAAGCCCATCTTCT

> -21

GTTCGCGTTCACGCTCATTATCTCT

> -8.8

GGTCCCTCACCAGACTCTTACTTCATACT

> +1

TGTGTCTGCTAAAACTCCGGGTTGTA

> +54

AGCAGTGAGTTCAGTGTGACATGCA

> +69

GGGAAGCAAAGCATCTCAGAACACTAG

> +73

GGTAGGACATACTGCAGAGGTAGAAGCA

> +93

GCAGTTCTCAGTCTGCAAGGTTCTGA

> +157

GGTAGGGAGTAAAGAAGAGGAGACCTGTA

> +174

CCTCAATCCAGACATCTGCCAGTCT

> +207

CCCCAGAAGTAGCACCAGATTCTCAT

> +219

TCTCCACGTTGGGTGAGTTGTGTAT

> +249

CGGACACCGTGAGAAAAACCTATGAAG

3. The mouse regions for ChIP experiments are shown below. The regions in uppercase represent oligonucleotides for PCR amplification.

>ZIM1 PROMOTER ([chr7:6648987+6649503](http://genome.ucsc.edu/cgi-bin/hgTracks?hgsid=320051991&db=mm9&position=chr7:6648987-6649503&hgPcrResult=pack)) 517bp CCCAGACCACTGTCCTCTACAGAGctgtgatagggtggggacccacccag catgcagacaccctgccctctcagtcctctcccgtgttcttgcctctcgt cctcgcgcttggttatccgtccgtccgcactcgcctccaggcaccgcagc ctgcggccacccggagtggaatgccgcgggatgactttcaaaacccaagt ccccagctctagggcaccgcccacaggactacatgtcccagaaggtctca cgccgtccagcctgaggctccaggctatggagcgcccagcacccggcaca gcgcgctgggacctgtgggaaatgtagtccccagcaagatgcgctcgcta taagcgttcctcagacgctctgcccttctgtggctccacttctagtacta tagagaaattgcgtctcctggtggaaaaatttccttcggtagtaaaattt ctaaattgatagaggccgatggtagaaaagggaaagatttttaaGATTAT

GTGATGACACGCTGAGG

>Peg3-promoter ([chr7:6682431-6683038](http://genome.ucsc.edu/cgi-bin/hgTracks?hgsid=320051991&db=mm9&position=chr7:6682431-6683038&hgPcrResult=pack)) 608bp GGTTCAGTGTGGGTGCACTAGACTgccgaccctggtcggggtgtgtgcgt agagtgctgtgctccgggaggtgagtcagccggccacctggctgctctgc agcatgcaccctcttagataccgtctgcagagttcagatggtgtttgggg tgcgttgccgcgggccaggggcggcagaccatatcacggctcccaagggt aactgacaaggctgcagactgcgccttcgggaagggggaatcaccacgga gcggccgtgttgccgcagggatgccatttaggtgacagggatttaaagag gtattctataggtccaggcctcggagcctcagggtcccgcgatcttaggg tcttagggctttaggtgtgaaccacccagaatcccgggagaatccaggct ccctgtctcatctgcgtggccagccacgcagatgatgcagaagtcctgtt acaagaccaccacagtggacatcagagtgccgtctctgagcaacctcttc tgtggtttcattttctttgctcagcaaagtctaagggctatgcggttaac ccgatcgccaggtcgtctggctggcagggtcttcgCAATCTAGCCATCTA

CCGCTAAG

>ECR18 ([chr7:6866575-6866805](http://genome.ucsc.edu/cgi-bin/hgTracks?hgsid=320052449&db=mm9&position=chr7:6866575-6866805&hgPcrResult=pack)) 231bp TGTGATTTCTCTGGAGACACAGacaacaaacatgttcctcatcttttgaa acaaacaagtttctatagtgcaaaccgtggagatcacatttcccaggggc ggacatacctaaaccatttgtcactggatcggctttccctgcagctaatc ccccctcaacagctgtgttggccgctgcggtatgtcaaactgcttctgac ccgcccggggCTTCTGCTGGCAGGCAGGCAG

>Zfp264-promoter ([chr7:6929371+6929740](http://genome.ucsc.edu/cgi-bin/hgTracks?hgsid=320052109&db=mm9&position=chr7:6929371-6929740&hgPcrResult=pack)) 370bp TATGTTGAGGTGAGGCTTCCATCttcctcatgaagaatattaagctacat tttctgttgtcttagaagacgatcttcgtaggcagtgtctaaaagggaca catcatgagatccgctgagtgctgggatccacacctttgcactgaccctt agatgggcaaaaatccctttgcttcccaagctttatttcactggctgaag aatgccagtctcacccctatctgtgagtcctttcccacaaactccatctt ctagatgcccccacatttgaaagaaactgggacttttaacgtctttcaca ctgggcgagacttacgcactgttagtcctgtcggacaccgtgagaaaaAC

CTATGAAGACCACATTACCC
